# Refractory Seborrheic Dermatitis Treated With a 532-nm Q-Switched Potassium Titanyl Phosphate (KTP) Laser: A Case Report

**DOI:** 10.7759/cureus.104932

**Published:** 2026-03-09

**Authors:** Virgilio Blandon, Krimhild Serrano, Ruby Poveda, Miguel Borge, Erick Correa

**Affiliations:** 1 Dermatology, Hospital Carlos Roberto Huembes, Managua, NIC

**Keywords:** 532 nm laser, potassium titanyl phosphate (ktp) laser, q-switched ktp, refractory dermatitis, refractory seborrheic dermatitis, seborrheic dermatitis, sedasi score

## Abstract

Seborrheic dermatitis (SD) is a common, chronic inflammatory skin disorder that can be challenging to manage when refractory to conventional topical therapies. This case report describes the use of a 532-nm Potassium Titanyl Phosphate (KTP) Q-switched laser in a patient with refractory SD. A 25-year-old male patient presented with persistent erythematous, scaly, and pruritic facial lesions. Clinical evaluation included histopathological confirmation and Seborrheic Dermatitis Area and Severity Index (SEDASI) scoring, with an initial score of 46 points.

The patient underwent four sessions of 532-nm KTP Q-switched laser treatment (8 J/cm², 10-ns pulse duration, 3-mm spot size, 5 Hz frequency, three non-overlapping passes per session), in combination with standard topical therapy (ketoconazole 2% shampoo and ceramide-based sunscreen). Following the fourth session, the SEDASI score decreased from 46 to 23 points, corresponding to a reduction from “very severe” to “moderate” disease severity. This was accompanied by clinical improvement in erythema, scaling, and pruritus. A three-month follow-up demonstrated sustained improvement without recurrence. No adverse effects were observed apart from transient erythema. Immediate whitening noted during treatment was consistent with a possible photomechanical effect.

In this single case, 532-nm KTP Q-switched laser therapy was well tolerated and associated with clinical improvement. Given the inherent limitations of a case report, these findings should be interpreted with caution. Further controlled studies are required to better define the safety, efficacy, and potential role of this modality in refractory SD.

## Introduction

Seborrheic dermatitis (SD) is a chronic, relapsing inflammatory disorder of sebaceous-rich skin, driven by a complex interplay of Malassezia colonization, sebum overproduction, and immune-barrier dysfunction [1-4]. Central to its pathophysiology are alterations in stratum corneum lipids, including ceramide profiles, which are key structural lipids involved in maintaining epidermal barrier integrity. Disruption of these lipids contributes to barrier dysfunction and increased transepidermal water loss, ultimately promoting the inflammatory manifestations of SD such as erythema, scaling, and pruritus [1-4]. Dermoscopy has refined our appreciation of the erythematous component, revealing underlying vascular abnormalities, such as atypical vessels and arborizing red lines, that correlate with disease severity [5]. These findings position aberrant cutaneous vasculature as a critical and addressable endpoint in SD management.

Current first-line therapies, primarily topical corticosteroids and antifungals, are often hampered by high relapse rates, tachyphylaxis, and potential side effects, with much of the supporting evidence derived from short-duration, underpowered trials [6,7]. While systemic antifungals are efficacious for moderate-to-severe non-scalp SD (Level of evidence 1A), their use is constrained by off-label status and concerns regarding systemic toxicity [6]. These limitations underscore a pressing need for novel, pathophysiology-driven treatment strategies.

Vascular-targeting laser therapies have been increasingly explored in inflammatory dermatologic conditions. Pulsed dye laser treatment, for example, has demonstrated therapeutic benefit in several inflammatory skin diseases, including psoriasis, acne, and rosacea, likely through modulation of superficial cutaneous vasculature and inflammatory pathways [8].

The 532 nm Potassium Titanyl Phosphate (KTP) laser represents a theoretically grounded candidate for targeting the vascular component of SD. The well-established affinity of 532 nm light for oxyhemoglobin within the superficial dermal plexus provides the foundational rationale for its use [9,10]. In this case, we utilized a Q-switched variant that delivers nanosecond-range pulses. This approach is supported by evidence showing that frequency-doubled, Q-switched Neodymium-doped Yttrium Aluminium Garnet (Nd:YAG) lasers (532 nm) can effectively treat cutaneous vascular lesions through photomechanical mechanisms, in which ultrashort pulses produce mechanical disruption of superficial vascular structures [11,12]. We hypothesize that this laser can effectively correct the vascular abnormalities linked to persistent erythema in SD.

While adjunctive therapies like ceramide-rich emollients and topical antifungals address barrier integrity and microbial load [3,4], the vascular component appears to be a relatively unexplored therapeutic target in current management strategies. This case report describes the novel application and clinical outcomes of the 532 nm KTP laser in a patient with refractory SD, advancing the hypothesis that direct modulation of vascular hyperactivity represents a promising therapeutic avenue where conventional therapies frequently fall short.

## Case presentation

**Figure 1:**
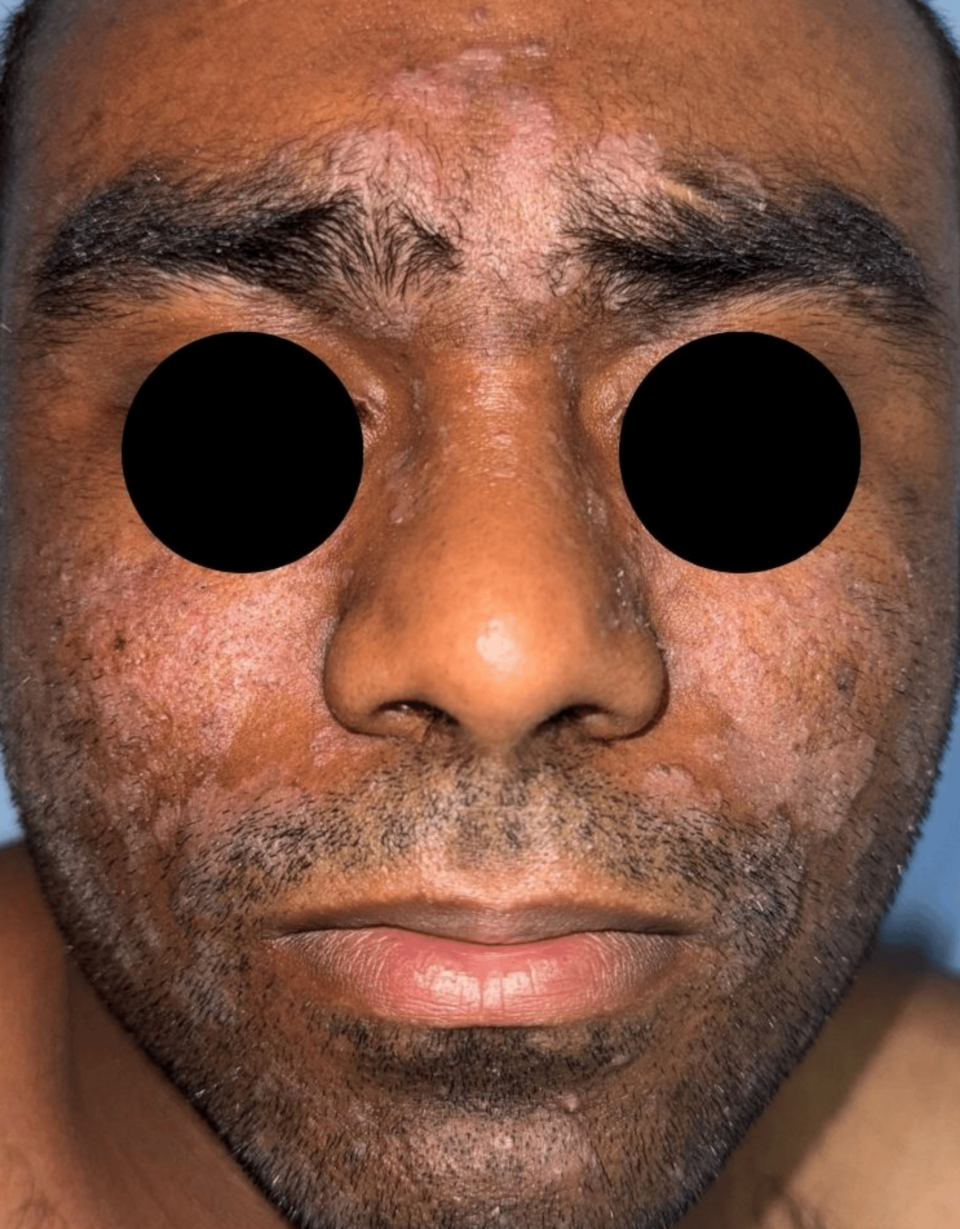
Clinical presentation of seborrheic dermatitis prior to treatment Erythematous, scaly plaques with greasy surface and ill-defined borders involving the glabella, eyebrows, malar region, nasolabial folds, mustache area, and beard. Patient consent for the publication of this image in an open-access online journal has been received.

A 25-year-old male patient presented with a two-year history of severe, refractory SD, manifesting as pruritic, erythematous plaques on the scalp, face, retroauricular regions, and chest (Figure 1).

Previous therapies, including topical corticosteroids (fluocinonide acetonide cream 0.05%, hydrocortisone cream 1%, both used intermittently for six months), systemic and topical antifungals (itraconazole 100 mg/day x two weeks, ketoconazole cream used daily for 10 weeks, and ketoconazole shampoo twice per week for two years), which was discontinued due to elevated liver enzymes (Alanine Aminotransferase: 183 U/L, Aspartate Aminotransferase: 177 U/L); emollients (daily for >one year), zinc pyrithione shampoo (intermittently for 1.5 years), and intermittently used coal tar and topical antibiotics. Despite adherence to these treatments, all these interventions failed to improve lesions, with persistent clinical signs and the patient's consistent report of a continued lack of clinical response and worsening symptoms. This continued lack of response, coupled with worsening symptoms, caused significant occupational and psychosocial distress.

**Figure 2:**
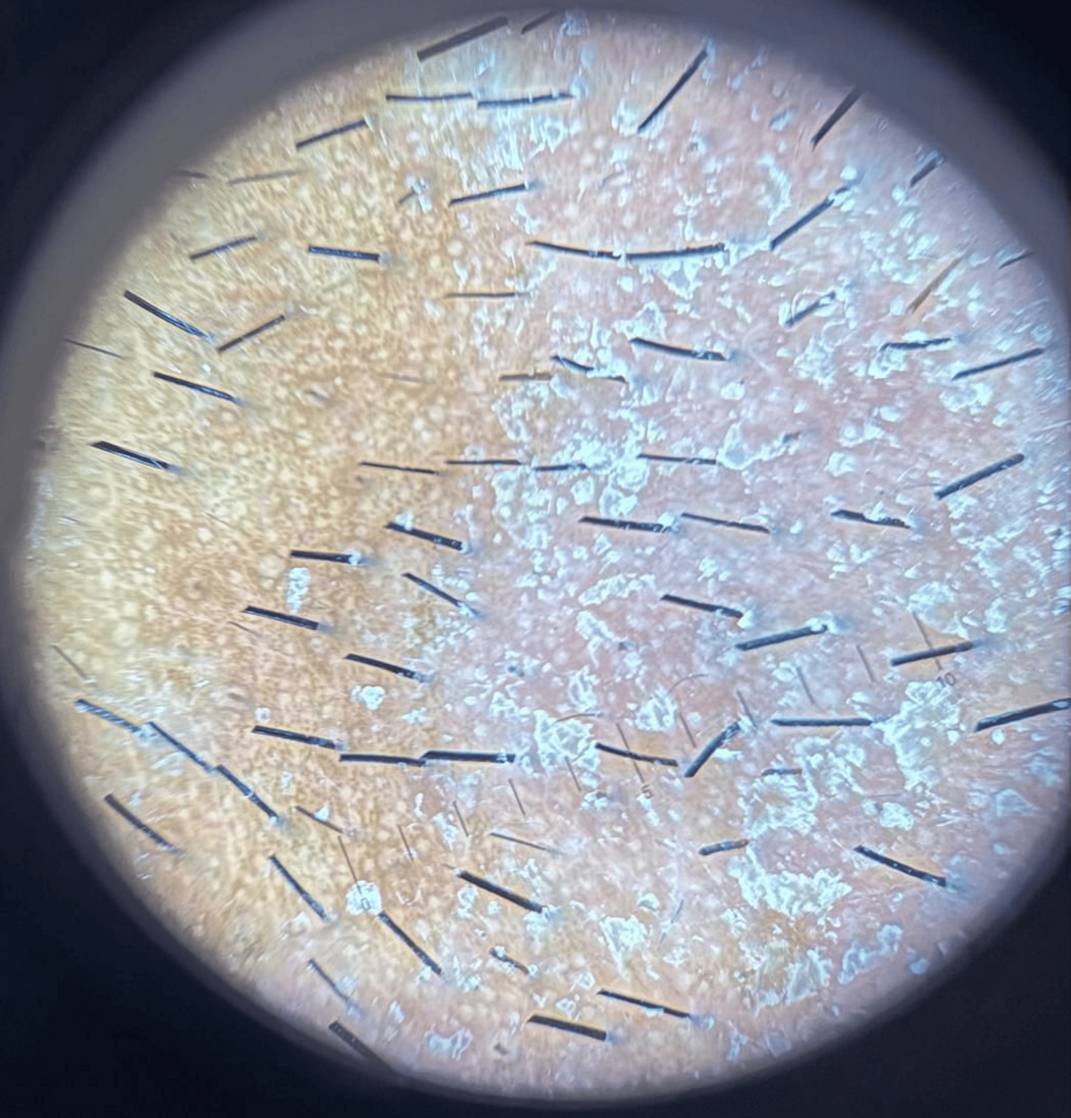
Dermoscopy of seborrheic dermatitis before treatment Polarized dermoscopic image reveals atypical vascular patterns consisting of multiple looped vessels, ring-like vessels, and dots, perifollicular yellowish halos (“oil-drop” sign), and background honeycomb-like pigmentation.

Physical examination revealed well‑demarcated yellowish‑white scaly plaques. Dermoscopic evaluation identified vascular patterns consisting of multiple looped vessels, ring-like vessels, and dots, set against a background of mild-to-moderate erythema. Additional findings included perifollicular scaling and honeycomb-patterned pigmentation (Figure 2).

**Figure 3:**
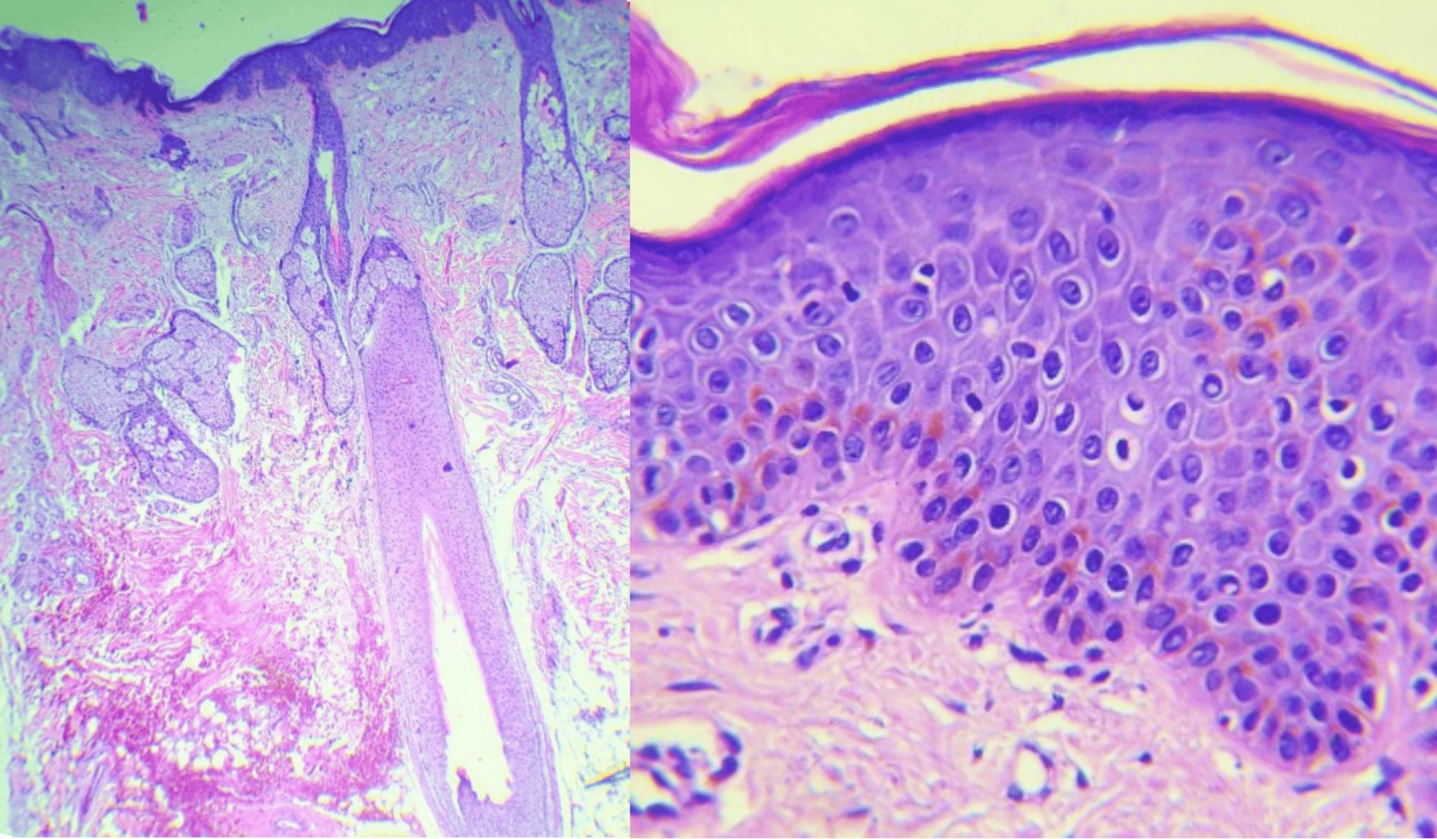
Photomicrographs of a skin biopsy from the patient Photomicrographs of a skin biopsy demonstrating histopathologic features consistent with seborrheic dermatitis. Low-power view (left) shows mild psoriasiform hyperplasia of the epidermis with focal spongiosis and prominent folliculosebaceous units surrounded by a superficial perivascular and perifollicular lymphocytic infiltrate. High-power view of the same biopsy (right) demonstrates mild epidermal spongiosis and dilated blood vessels in the papillary dermis, with a sparse inflammatory infiltrate and no significant epidermal acanthosis or parakeratosis in this field. Hematoxylin and eosin stain; original magnifications ×20 (left) and ×40 (right)

Malar biopsy confirmed superficial perivascular dermatitis with spongiotic and psoriasiform patterns, excluding pemphigus and lupus (Figure 3).

The potassium hydroxide examination was positive for hyphae. Serological testing for HIV was negative. Taken together, these clinical, dermoscopic, mycological, and histopathological findings supported the diagnosis of severe SD.

Facial involvement was subsequently assessed using the Seborrheic Dermatitis Area and Severity Index (SEDASI), a facial-specific severity scale validated by Micali et al. The baseline SEDASI score was 46 points, corresponding to very severe disease (Table 1) [13]. 

**Table 1:** Seborrheic Dermatitis Area and Severity Index (SEDASI) Facial evaluation using the Seborrheic Dermatitis Area and Severity Index [13]. The face is divided into 4 regions: nose, forehead, left cheek, and right cheek. Each region is assessed based on 4 parameters: (one) lesion extension (0 = no lesions to 6 = 90-100%), (two) presentation pattern (0 = none to 3 = large/confluent patches), (three) erythema degree (0 = none to 3 = intense red), and (four) scaling degree (0 = none to 3 = large/thickened scales). The total SEDASI score ranges from 0 to 60. Global severity is categorized as: mild (1 to 14), moderate (15 to 29), severe (30 to 44), and very severe (≥45). SEDASI scoring was performed by the treating dermatologist during clinical evaluation and was not blinded.

Facial Region	Item	Initial Score (Baseline)	Final Score (Week 8)
Nose (including nasolabial folds)	Extension of lesions	3	2
Nose (including nasolabial folds)	Presentation pattern	2	1
Nose (including nasolabial folds)	Erythema degree	2	1
Nose (including nasolabial folds)	Scaling degree	2	1
Forehead (including eyebrows and upper eyelids)	Extension of lesions	2	1
Forehead (including eyebrows and upper eyelids)	Presentation pattern	3	2
Forehead (including eyebrows and upper eyelids)	Erythema degree	3	1
Forehead (including eyebrows and upper eyelids)	Scaling degree	3	1
Left cheek (including lower eyelids, ear, and chin)	Extension of lesions	4	2
Left cheek (including lower eyelids, ear, and chin)	Presentation pattern	3	2
Left cheek (including lower eyelids, ear, and chin)	Erythema degree	3	1
Left cheek (including lower eyelids, ear, and chin)	Scaling degree	3	1
Right cheek (including lower eyelids, ear, and chin)	Extension of lesions	4	2
Right cheek (including lower eyelids, ear, and chin)	Presentation pattern	3	2
Right cheek (including lower eyelids, ear, and chin)	Erythema degree	3	1
Right cheek (including lower eyelids, ear, and chin)	Scaling degree	3	1
Total	—	46	23

The patient received four biweekly 532 nm Q-switched KTP laser sessions (8 J/cm², 5 Hertz, spot size 3 mm, 10-ns pulse duration, three laser passes), combined with ketoconazole 2 % shampoo and ceramide-rich sunscreen. The treatment course lasted eight weeks, with clinical assessments performed at baseline and at each treatment visit. Transient whitening was consistently observed immediately after each laser pulse, a recognized clinical sign of intravascular photomechanical effect [14]. This observed phenomenon served as the real-time clinical endpoint during treatment. Transient post-treatment erythema was reported by the patient to last an average of one day following each session. Notably, no purpura was observed at any point during or after the treatments, despite the use of parameters within the known efficacious range for vascular lesions. 

**Figure 4:**
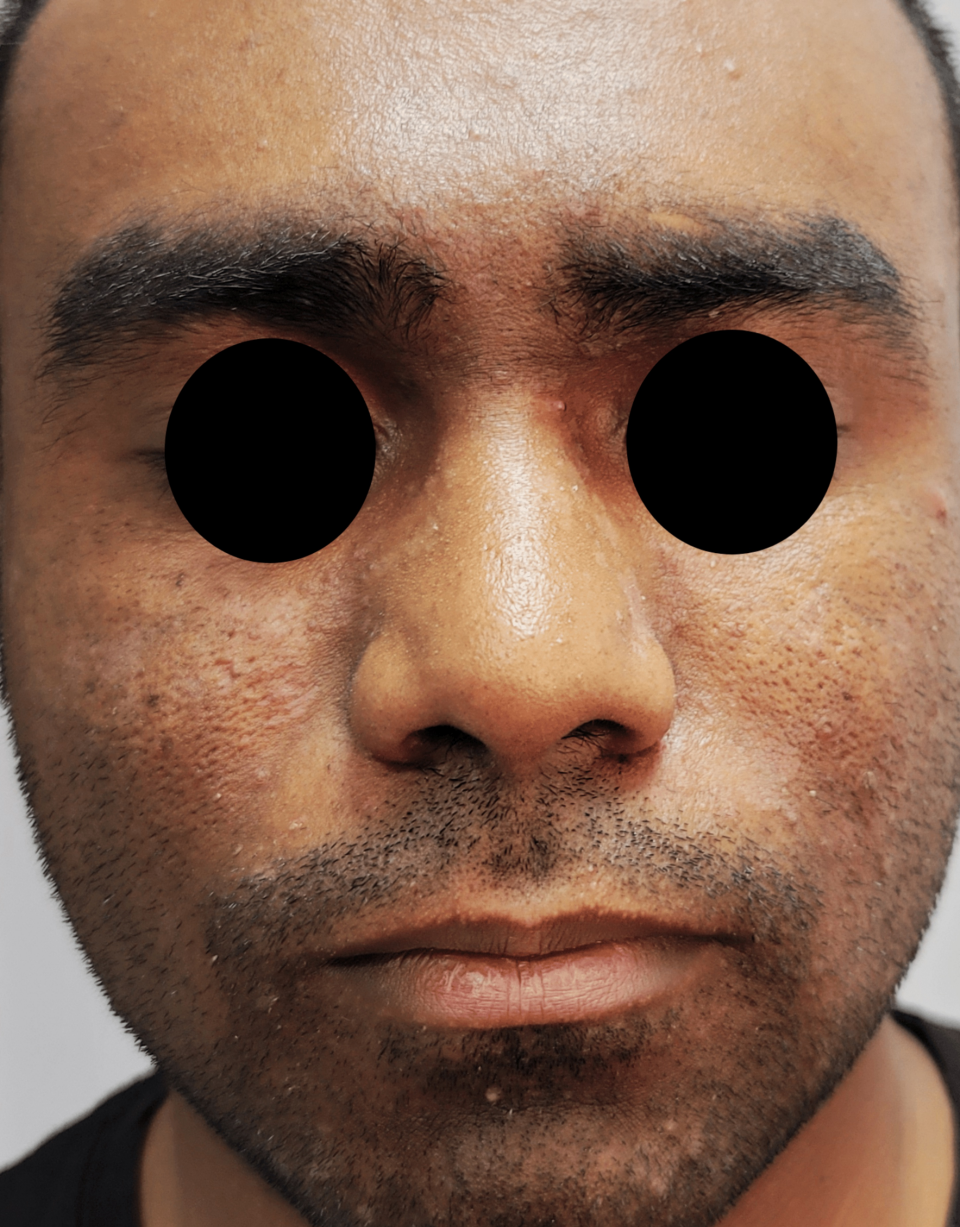
Clinical presentation of seborrheic dermatitis after treatment Marked reduction of erythema and scaling across previously affected areas, including the glabella, eyebrows, malar region, nasolabial folds, mustache area, and beard. Also an overall improvement in skin tone is observed, with no evidence of post-treatment hypopigmentation or hyperpigmentation following potassium titanyl phosphate (KTP) laser therapy. Patient consent for the publication of this image in an open-access online journal has been received.

By week eight (following the fourth session), the SEDASI score decreased from 46 (very severe) at baseline to 23 (moderate), indicating a substantial reduction in disease severity (Figure 4).

**Figure 5:**
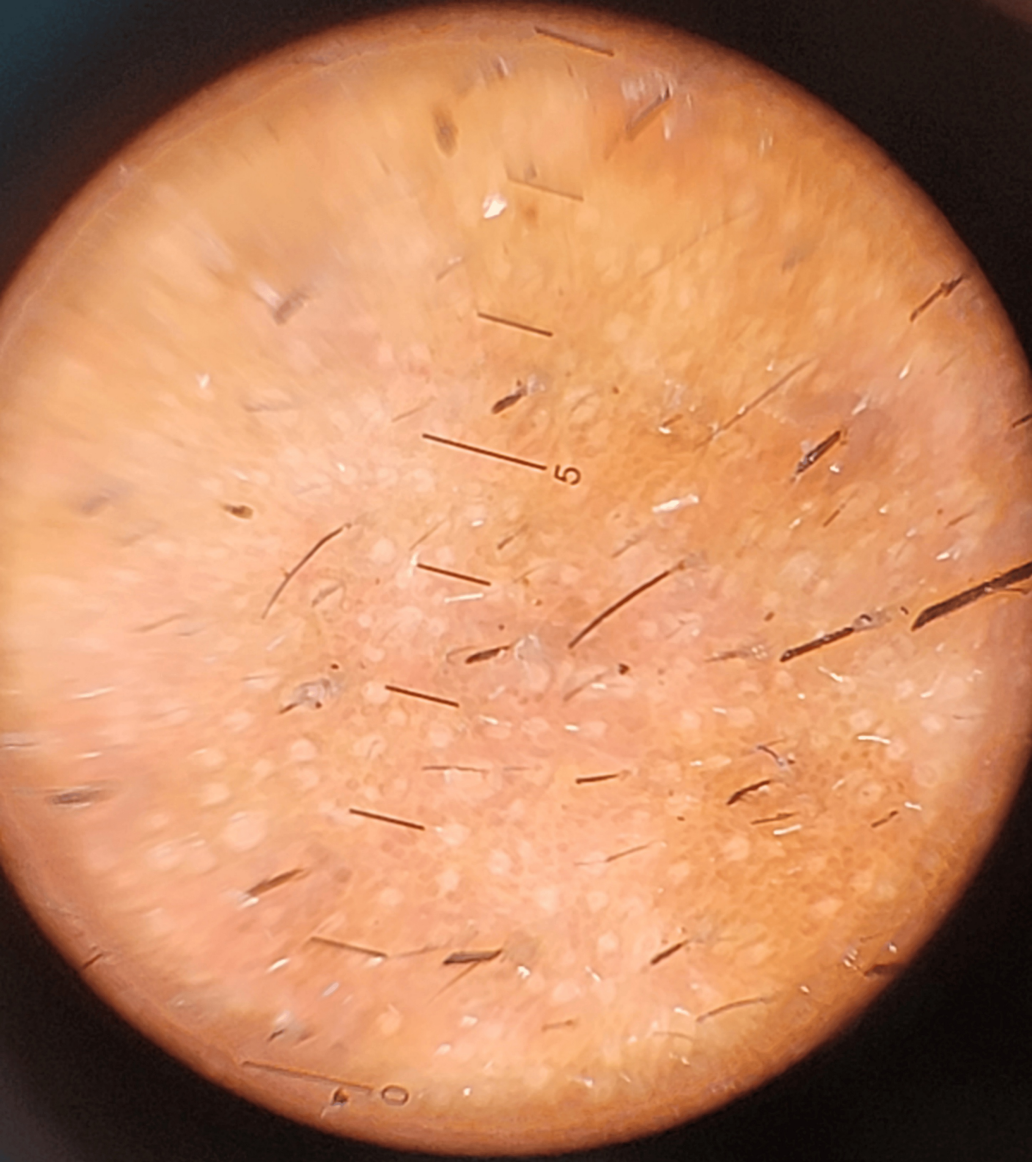
Dermoscopy of seborrheic dermatitis after treatment Post-treatment dermoscopic image demonstrating improvement in the vascular pattern and perifollicular yellowish halos, with a marked reduction in scaling and background pigmentation.

Post-treatment dermoscopy revealed improved vascular patterns and reduced scaling (Figure 5).

Only transient post-treatment erythema occurred, and remission persisted at three months of follow-up.

## Discussion

Our patient achieved a 50% SEDASI reduction and a notable dermatoscopic improvement, suggesting the significant impact of vascular-targeted laser therapy in this case of refractory SD. The rationale for using the 532 nm KTP laser was twofold. First, it was the available vascular laser system in our institution. Second, its well-documented ability to selectively target oxyhemoglobin in dilated superficial vessels provided a sound pathophysiological basis for addressing the vascular component of SD [9-12], as documented by dermoscopy.

Significantly, our literature search across PubMed, Google Scholar, and ClinicalTrials.gov (conducted up to February 19, 2026) did not identify prior clinical trials or case reports specifically evaluating the 532-nm KTP laser for SD. To the best of our knowledge, this suggests that the present report may represent one of the earliest documented clinical applications of this specific laser modality for SD.

Our PubMed search strategy included: (("seborrheic dermatitis"[MeSH Terms] OR "seborrheic dermatitis"[All Fields] OR "SD"[All Fields]) AND ("KTP laser"[All Fields] OR "532 nm laser"[All Fields] OR "532nm laser"[All Fields] OR "532 nm Nd:YAG laser"[All Fields] OR "532nm Nd:YAG laser"[All Fields])), yielding 31 results, none of which were relevant upon review.

For Google Scholar, an advanced search conducted on February 19, 2026 used the following parameters: 'with all of the words': KTP laser, 532 nm laser, 532nm laser, 532 nm Nd:YAG laser, and 532nm Nd:YAG laser; 'with the exact phrase': KTP laser; and 'with at least one of the words': "KTP laser", "532 nm laser", "seborrheic dermatitis", "dermatitis seborreica", and SD. This search yielded 208 results, none describing the use of the KTP laser for SD.

In the case of ClinicalTrials.gov, searches were performed using the fields 'Condition/disease' (seborrheic dermatitis) and 'Intervention/treatment' (laser therapy), which yielded no related studies.

Taken together, these findings suggest that the use of the 532-nm KTP laser for SD has not been previously described in the available literature, highlighting the potential novelty of this observation.

The selection of the specific laser parameters was guided by existing literature on Q-switched 532 nm lasers. While Goldberg et al. reported efficacy in vascular lesions using lower fluences (3-4 J/cm²), their system employed a shorter 5-ns pulse duration [11]. Given that our laser system had a fixed, longer pulse duration of 10 ns, we reasoned that a higher fluence would be necessary to achieve a similar photomechanical effect. Therefore, we selected a fluence of 8 J/cm², aligning with the effective range (4-12 J/cm²) reported by Cisneros et al. for a laser with a comparable 10-ns pulse duration, aiming to balance efficacy with an optimal safety profile [12]. Critically, Cisneros et al. did not report purpura as a side effect, a finding consistent with our own clinical observations and a key consideration for our safety profile [12].

The rationale for utilizing the KTP laser, despite the lack of direct precedent in SD, stems from its established success in other vascular-driven dermatoses [9-12]. This laser's 532 nm wavelength offers high oxyhemoglobin absorption, enabling precise targeting of the superficial dermal plexus [9,10]. The choice of a Q-switched, nanosecond-pulse laser was strategic. For microvessels, such as the dilated capillaries documented in SD [15,16], nanosecond pulses achieve stress confinement, a condition where the pulse duration is shorter than the acoustic transit time across the target [17]. This physical principle underpins a photomechanical mechanism of action, leading to precise vessel disruption via photoacoustic injury, a process that minimizes thermal diffusion [11,12,17]. This represents a fundamental distinction from longer, millisecond-domain pulses, where the goal is thermocoagulation and intravascular cavitation is often an undesirable event associated with vessel rupture and purpura [14]. In our application, for nanosecond-domain pulses, the photomechanical cavitation effect is the primary intended mechanism. The consistent observation of immediate, transient whitening during treatment is the clinical hallmark of this process, further corroborating our mechanistic rationale [14].

In the present case, the patient's marked improvement is best explained by the introduction of laser therapy. While ketoconazole shampoo and ceramide-rich sunscreen were used concomitantly, it is critical to note that these agents constituted the patient's long-term, baseline regimen which had previously proven insufficient. The significant reduction in erythema and the rapid improvement of vascular patterns on dermoscopy following laser sessions strongly suggest that the laser's effect on the pathological vasculature was the pivotal therapeutic intervention. The temporal correlation between laser application and clinical improvement, in a context where other variables remained constant, points to a primary effect of the laser.

This report has several limitations, inherent to its design as a single case study. The lack of a control group and the inability to completely isolate the effect of the laser from the concomitant topical therapies prevent definitive conclusions about its efficacy in isolation. Furthermore, the follow-up period, while showing sustained improvement, is relatively short for a chronic condition like SD. The mechanistic interpretation, while supported by established laser physics and clinical observation, would be strengthened by direct capillaroscopic and histological correlation in future studies.

Notwithstanding these limitations, our findings provide a strong rationale for further investigation. Randomized controlled trials are warranted to definitively establish the efficacy of the 532 nm Q-switched KTP laser for refractory SD. Such studies should aim to determine optimal laser parameters (fluence, pulse duration, spot size), treatment frequency, and long-term outcomes. Future research should also seek to quantify patient-reported quality-of-life improvements and assess the cost-effectiveness of this approach compared to existing systemic therapies.

## Conclusions

This reported case suggests that the 532-nm Q-switched KTP laser may be associated with meaningful and sustained improvement in a patient with refractory SD. The temporal relationship between laser application and clinical response, together with the observed whitening consistent with a photomechanical effect, supports the hypothesis that targeting dermal vasculature could be relevant in SD. As a proof-of-concept, hypothesis-generating observation, this well-tolerated approach, using a widely available laser platform, may represent a promising therapeutic option for refractory cases. However, its efficacy and safety require confirmation in appropriately designed randomized controlled trials.
